# Diabetes mellitus in a patient with glycogen storage disease type Ia: a case report

**DOI:** 10.1186/s13256-017-1462-5

**Published:** 2017-11-12

**Authors:** Aviva Cohn, Anupam Ohri

**Affiliations:** Department of Internal Medicine, Rutgers Robert Wood Johnson Medical School, 1 RWJ Place, MEB 486 PO Box 19, New Brunswick, NJ 08903 USA

**Keywords:** Diabetes, Glycogen storage disease, Hyperglycemia, Hypoglycemia, Metabolic syndrome

## Abstract

**Background:**

Glycogen storage disease type Ia is a genetic disorder that is associated with persistent fasting hypoglycemia and the inability to produce endogenous glucose. The development of diabetes with glycogen storage disease is exceedingly rare. The underlying pathogenesis for developing diabetes in these patients is unclear, and there are no guidelines for treatment.

**Case presentation:**

We describe a case of a 34-year-old woman of South Asian descent with glycogen storage disease type Ia, who developed uncontrolled diabetes mellitus as a young adult. Hyperglycemia was noted after childbirth, and worsened years later. Treatment for diabetes was difficult due to risks of hypoglycemia from her underlying glycogen storage disease. With minimal hypoglycemic events, the patient’s blood glucose improved with exercise in combination with a sodium-glucose co-transporter 2 inhibitor and an alpha glucosidase inhibitor.

**Conclusion:**

We report a rare case of diabetes in the setting of glycogen storage disease-Ia. Based on the literature, there appears to be a relationship between glycogen storage disease and metabolic syndrome, which likely plays a role in the pathogenesis. The management of glycemic control remains a clinical challenge, requiring management of both fasting hypoglycemia from glycogen storage disease, as well as post-prandial hyperglycemia from diabetes mellitus.

## Background

Glycogen storage disease type 1 (GSD-I) is a rare genetic condition that develops due to an inborn error of metabolism causing deficient activity of the enzyme, glucose 6-phosphatase [1]. This enzyme is essential to hydrolyze glucose-6 phosphate (G6P) into biochemically active glucose, the final step of gluconeogenesis and glycogenolysis. Lack of this enzyme manifests clinically as hypoglycemia in the postabsorptive state, as well as complications from excess buildup of glycogen and G6P resulting in organ dysfunction including hepatomegaly and kidney disease [2].

**Fig. 1:**
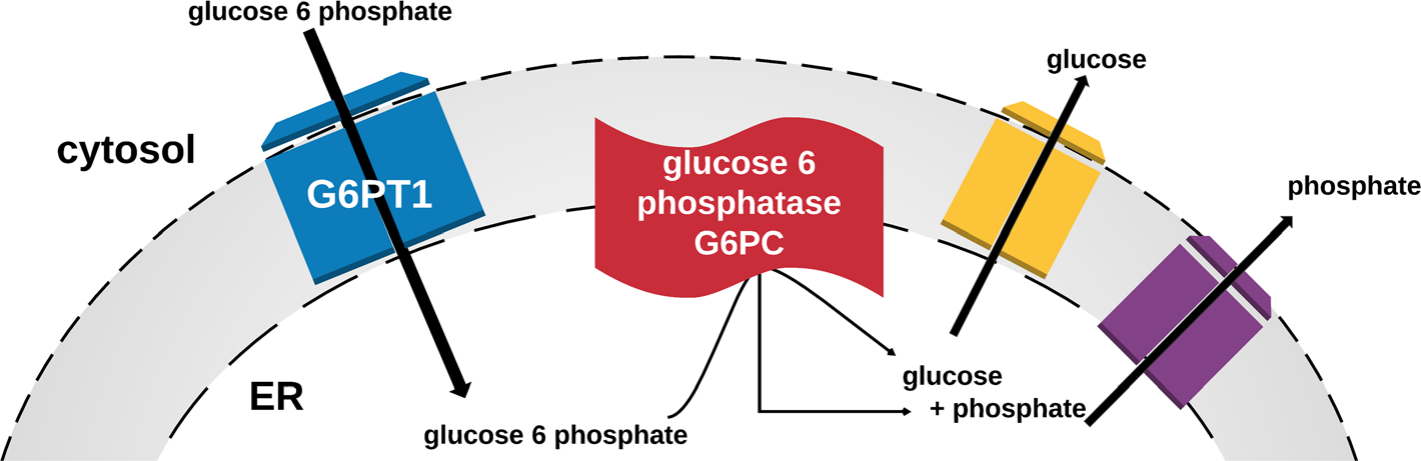
Enzyme deficiencies in glycogen storage disease type 1a vs glycogen storage disease type 1b [15]. In the final step of endogenous glucose production, glucose-6 phosphate enters the endoplasmic reticulum of the cell through a transporter known as G6PT. Upon entering the endoplasmic reticulum the enzyme glucose-6 phosphatase, also known as G6PC, hydrolyzes glucose-6 phosphate into glucose and inorganic phosphate. Glucose then leaves the endoplasmic reticulum and is released systemically. In glycogen storage disease type 1a there is a mutation of the enzyme G6PC resulting in a buildup of glucose-6 phosphate in the endoplasmic reticulum. In glycogen storage disease 1b, there is a mutation of G6PT, whereby glucose-6 phosphate cannot enter the endoplasmic reticulum to undergo the catalytic reaction. G6PT1: glucose 6 phosphate translocase 1, G6PC: glucose 6 phosphatase catalytic subunit, ER: Endoplasmic Reticulum

GSD-I is inherited in an autosomal recessive manner, with an incidence of 1 out of 100,000 births [1, 3]. There are two subtypes of GSD-I, known as GSD-Ia and GSD-Ib, which are distinguished by the enzymatic deficiency (see Fig. 1). This manuscript will focus on GSD-Ia which is the more common subtype, for which there is a deficiency of the enzyme G6PC.

Patients with GSD-Ia are recognized early for their episodes of hypoglycemia in childhood which can be severe, causing seizures, neurocognitive decline, as well as stunted growth [4]. Treatments such as uncooked cornstarch or continuous overnight gastric feedings are used to ensure there is sufficient glucose in the fasting state [1]. Long-term complications of the disorder include liver adenomas and steatosis, kidney dysfunction, lactic acidosis, elevated uric acid, and profound hypertriglyceridemia [4].

GSD-Ia is a disorder that highlights the importance of glucose as a basic energy source throughout the body. Understanding the physiology of uptake, utilization, and storage of glucose is essential for medical treatment of various pathologies involving altered glucose metabolism. Patients with GSD-1a suffer from fasting hypoglycemia; thus it would seem paradoxical for an individual who has primarily a disease of decreased endogenous glucose production to develop diabetes. We describe a case of a patient with known GSD type Ia who developed uncontrolled diabetes. In reviewing our patient’s case presentation, we explore the pathophysiology of glucose homeostasis, and the challenge to find an appropriate medical therapy.

## Case presentation

A 34-year-old woman of South Asian descent with a past medical history of obesity [body mass index (BMI) 41.6], polycystic ovarian syndrome, psoriasis, horseshoe kidney with uric acid kidney stones, and hypothyroidism, was diagnosed at age 3 with GSD-Ia. As an infant she had hypoglycemic episodes resulting in seizures. A liver biopsy showed decreased G6PC enzymatic activity of 0.45 μmol/min/g tissue (N 3.50 ± 0.8 μmol/min/g tissue) with increased glycogen content. Gene analysis revealed a mutation of the G6PC gene homozygous for 50delGT, thus confirming the diagnosis of GSD-Ia.

Initially, our patient was managed with frequent feedings and by the age of 9, a night-time cornstarch regimen was initiated to prevent hypoglycemia. She had expected complications of the disease, including uric acid kidney stones, lactic acidosis, hepatic adenomas with hepatomegaly, and diffuse steatosis.

At the age 31 years, our patient became pregnant and did not undergo routine gestational diabetes screening due to her underlying GSD. Three days prior to delivery, she was incidentally noted to be hyperglycemic for the first time, with a blood glucose more than 300 mg/dL (N 70–140 mg/dL). The elevated glucose was attributed to gestational diabetes for which she was started on insulin. Treatment was complicated by severe hypoglycemia with a blood glucose of 32 mg/dL, and she subsequently refused all forms of insulin.

Thereafter, our patient was lost to follow-up for 2 years. At the age of 33 years, she sought care when she had continued hyperglycemia and a hemoglobin A1c (HbA1c) of 13% (N 4.3–6.0%). On social history, our patient was working at an office job; she denied any toxic habits including smoking or alcohol use. There was no family history of consanguinity nor birth defects; her young child was healthy. She had a maternal grandmother with diabetes, and her mother had prediabetes. On examination, our patient had a BMI of 41.6, short stature, and generalized obesity. Her heart and lung examinations were unremarkable; she had an obese abdomen with hepatomegaly. A skin examination was notable for acanthosis nigricans, with scattered psoriatic patches. Neurologically, she was alert and appropriate, and her examination was non-focal. Laboratory tests were significant for elevated triglycerides of 960 mg/dL, with microalbuminuria, and otherwise normal liver function tests. Magnetic resonance imaging (MRI) of her abdomen showed multiple liver adenomas with diffuse steatosis. Further investigation of her diabetes showed an insulin level of 32 μIU/mL (n 1.9–23 μIU/mL), and a high c-peptide of 5.2 ng/mL (N 0.8–3.1 Ng/mL), with a fasting blood glucose of 311 mg/dL (N 70–140 mg/dL). A homeostatic model assessment of insulin resistance (HOMA IR) calculation to determine insulin resistance was calculated as 24.6 (N <2.5), thus indicating severe insulin resistance [5]. Despite the diagnosis of diabetes, our patient continued her regimen of 5 tablespoons (tbsp) of uncooked cornstarch at night to prevent hypoglycemia from her underlying GSD.

With initial lifestyle modifications, including a lower carbohydrate diet, exercise, and attempting weight loss, our patient’s HgA1c improved from 13% to 11.9%. It was clear medication initiation was needed, however this was a challenge due to the patient’s underlying chronic liver disease, lactic acidosis, and refusal of insulin. Due to liver dysfunction, a sodium-glucose co-transporter 2 (SGLT2) inhibitor, a renally cleared medication, was trialed first. Our patient was treated with canagliflozin, with uptitration of the dosage to 300 mg daily. She concomitantly decreased her bedtime cooked cornstarch from 5 tbsp to 3 tbsp. With these interventions, after a few months, her HgA1c improved from 11.9 to 9% without episodes of hypoglycemia but notable elevation in lactate levels from 3.2 to 5.6 mmol/L (N 0.6–2.5 mmol/L).

**Fig. 2:**
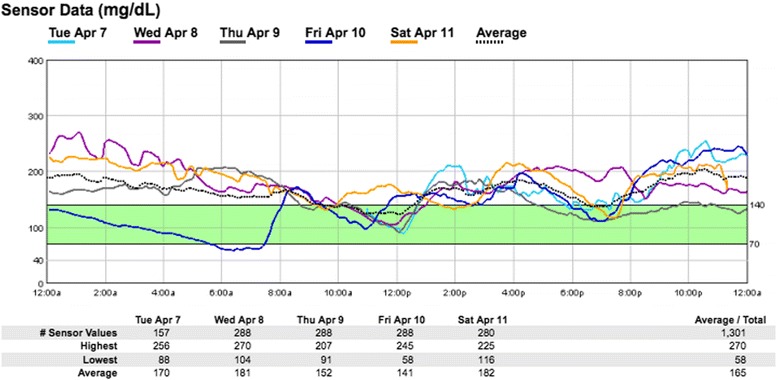
Continuous glucometer monitoring system after initiation of acarbose: Our patient’s continuous glucometer monitoring system details daily blood glucose trends from April 7 to 11, 2015 after starting treatment with acarbose 50 mg three times daily with meals. Blood glucose is represented on the y-axis, and hours of the day are on the x-axis. Each day is color-coded. The blood glucose appears to trend downward after midnight. There is one hypoglycemic episode noted on Friday April 10 at 7 a.m. There is another blood glucose trough at noon post exercise. Postprandial blood glucose elevation is seen at 2 p.m. and 9 p.m.

For more effective glycemic control, an alpha glucosidase inhibitor was added. A dose of acarbose 50 mg was started three times a day with meals. A continuous glucometer monitoring system (CGMS) 1 week after starting acarbose showed downtrending night-time blood glucose with an episode of hypoglycemia in the morning (see Fig. 2). Omission of acarbose at dinner-time effectively eliminated fasting hypoglycemia. Overall, it took approximately 1 year to control her blood glucose, and at the age of 34 years, her diabetes was at goal, at which point our patient was concomitantly able to lose 11 lbs. A combination of canagliflozin, acarbose, and weight loss decreased her starting HgA1c from 13 to 6.5%. HOMA IR was reduced from 24.6 to 1.9, indicating significant improvement in insulin sensitivity.

## Discussion

Typically, patients with GSD-Ia struggle with persistent episodes of fasting hypoglycemia; our case of a patient with GSD-Ia and type 2 DM is rare. The paradoxical nature of the two conditions presents itself as a challenge to glucose homeostasis.

There are few case reports in the literature of patients with different subtypes of GSD who later developed DM. Most of these reports describe patients who develop DM in the setting of pancreatic insufficiency and insulinopenia, which does not appear to be the sole etiology in our patient with insulin resistance [4, 6]. A literature search revealed the only known cases of GSD-Ia and type 2 DM are four patients mentioned in a poster presentation at a clinical meeting. These cases differ in that three of the four patients were pediatric age, and for all cases described, glycemic control was achieved purely with weight reduction [7]. In our case, the patient was an adult, and despite rigorously attempting weight loss, she ultimately required additional medical therapy to achieve glycemic control.

In determining the etiology of diabetes in our patient there are several proposals. Typically, patients with GSD-Ia have <10% of normal functioning G6PC enzymatic activity. There is, however, a spectrum of enzymatic dysfunction; while some patients have no functional G6PC, others might have partial [8]. In our case, it is plausible that with some functional enzymatic activity (12% of normal), combined with obesity and a lifestyle of a high carbohydrate diet, our patient developed insulin resistance which progressed to overt diabetes.

Additionally, one study demonstrated that patients with GSD-I have an uncharacteristic insulin response to food when challenged with a glucose tolerance test. Subjects were adults with GSD-I subtypes who ingested a glucose load, and post-prandial insulin levels were measured. The results showed that these patients mounted a delayed insulin response to food, resulting in post-prandial elevated blood glucose as compared to controls [6]. A dysregulation, or overload of this mechanism, with excess carbohydrates and weight gain could explain the disproportionately elevated post-prandial levels of blood glucose evident in our case.

Recent literature describes a possible correlation between GSD-Ia and the development of metabolic syndrome, which could explain the propensity for our patient to develop diabetes. Melis *et al*. showed that in a cohort of patients with GSD-Ia, there was a statistically significant increase in the features of metabolic syndrome when compared to those with GSD-Ib and controls [5]. There was evidence of greater insulin resistance, as well as hypertriglyceridemia, and increase in waist circumference in the GSD-Ia group. The proposed biochemical mechanism is that due to the deficiency of G6PC there is an excess of the substrate G6P in the endoplasmic reticulum, as it cannot be properly hydrolyzed. The presence of excess G6P upregulates the activity of another enzyme, 11ß-hydroxysteroid dehydrogenase type 1 (11BHSD1), that is involved in the activation of glucocorticoids, and which has been previously thought to contribute to metabolic syndrome [5]. In this study, however, despite insulin resistance, there was no hyperglycemia described.

**Fig. 3:**
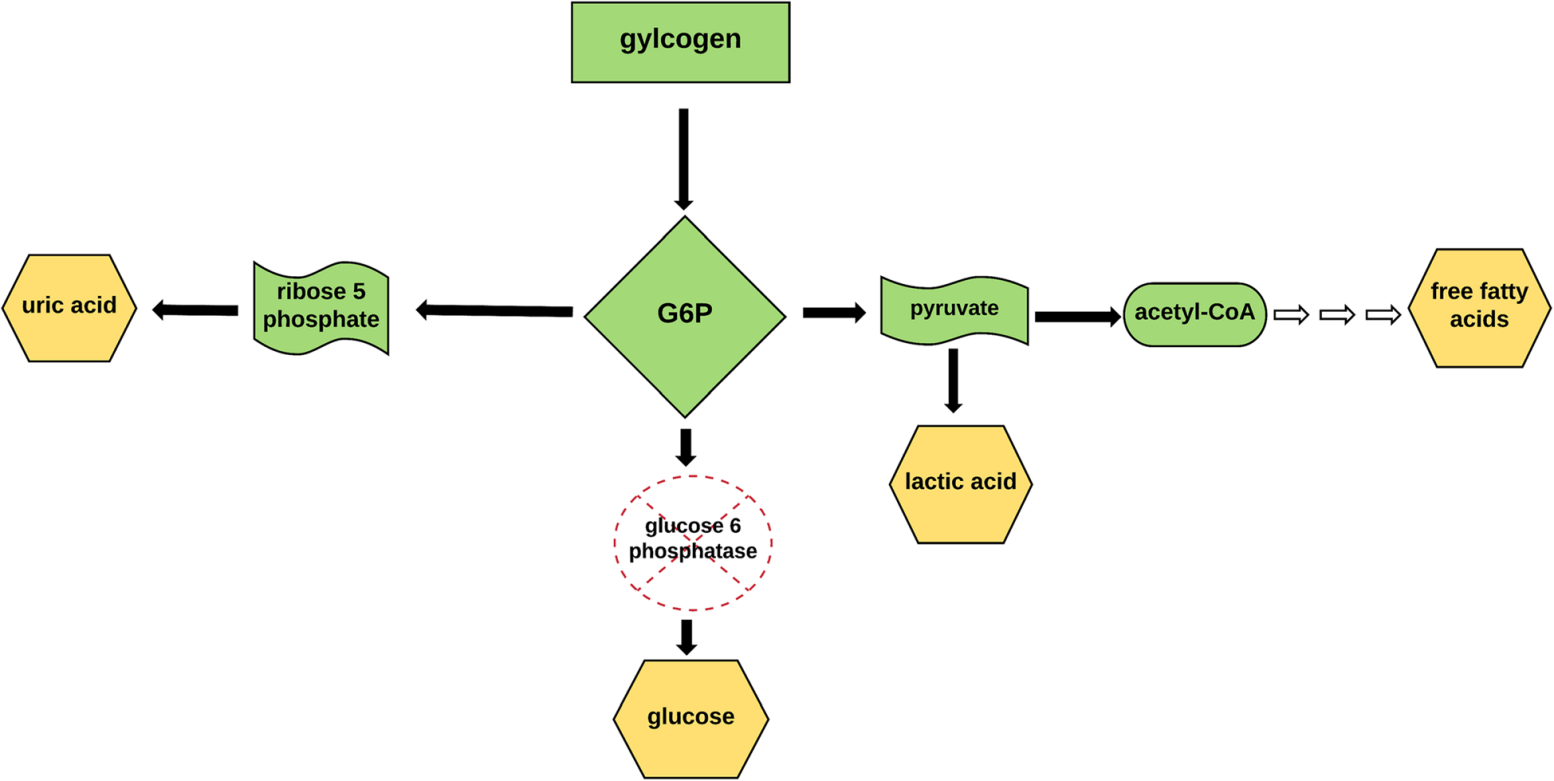
Alternative pathways for glucose-6 phosphate leads to lactic acidosis, hyperuricemia, and hyperlipidemia: In glycogen storage disease type 1a, there is deficient glucose-6-phosphatase, which results in the shunting of the substrate glucose-6-phosphate away from glucose, into alternative pathways. This results in elevated lactic acid, hyperuricemia, and hyperlipidemia. Source: Froissart R, *et al.* [3]

In addition, the very nature of GSD-Ia itself with hallmarks of hypertriglyceridemia and liver steatosis likely contributed to our patient’s insulin resistance. In GSD-Ia there is excess build up of the substrate G6P which shunts into other metabolites including free fatty acids and triglycerides (see Fig. 3). This production of excess free fatty acids causes an imbalance, whereby lipid supply to the liver exceeds lipid use, which plays a role in the pathophysiology of liver steatosis [9]. The injured fatty liver leads to altered cell-signaling pathways, which brings rise to insulin resistance [10]. Without the liver sensing insulin, there is decreased hepatic glucose uptake from the portal vein after a meal, and pronounced post-prandial hyperglycemia [11].

Our patient presented as a dilemma for treatment, requiring modulation of both fasting hypoglycemia from her GSD, as well as post-prandial hyperglycemia secondary to her diabetes. No guidelines exist on how to treat hyperglycemia in patients with GSD-Ia, and while insulin is an obvious option, it can exacerbate hypoglycemia. The use of a diagnostic CGMS on our patient was an effective tool that provided important information about general trends of blood glucose. The patient’s CGMS highlights her pattern of blood glucose trending down in the early morning and post-prandial hyperglycemia.

Exercise in our patient modestly improved glycemic control, due to its beneficial effects on insulin sensitivity and hyperglycemia, however this was not enough [12]. Medical therapy with canagliflozin improved our patient’s blood glucose in conjunction with weight loss. However, while patients with GSD already have a propensity for lactic acidosis, there is an additional risk of euglycemic diabetic ketoacidosis with the use of an SGLT2 inhibitor [13]. This potential side effect could worsen our patient’s existing metabolic acidosis, and therefore the use of an SGLT2 inhibitor requires careful monitoring. Acarbose was selected as a drug of choice in our patient, based on its mechanism in decreasing gastrointestinal absorption of complex carbohydrates. It is especially effective for improving post-prandial hyperglycemia, hyperinsulinemia, and hypertriglyceridemia [14]. While acarbose has a lower risk of hypoglycemia than insulin, caution must be used as it can prevent absorption of carbohydrates resulting in refractory hypoglycemia with oral intake of complex carbohydrates. In our case, the combination of acarbose and an SGLT2 inhibitor with weight loss provided glycemic control.

GSD-Ia remains a rare disorder; however, the life span of patients with GSD will expectantly become longer with better recognition and medical treatment. This unusual presentation of diabetes in a patient with GSD may become more frequently encountered as these patients enter adulthood, and understanding the pathophysiology to provide effective treatment will be necessary.

## Conclusions

GSD-Ia is a rare disorder which manifests with hypoglycemia and the inability to produce endogenous glucose. Despite a correlation between metabolic syndrome and GSD-Ia, there are few cases of patients with diabetes and GSD-Ia in the literature. This case reviews the pathophysiology of the metabolic disorder and highlights the rare presentation of a woman with GSD-Ia, who presented with diabetes. The challenges of treatment include worsening hypoglycemia from insulin, as well as potential kidney and liver dysfunction from GSD-Ia, which limits medication use. Exercise and diabetic medications including an alpha glucosidase inhibitor and an SGLT2 inhibitor, were the therapeutic choices used for our patient. With the increasing lifespan of GSD patients, this therapeutic challenge will likely present itself more often. Our case emphasizes the need for a discussion of the pathophysiology of the disease, and the risks versus benefits of different treatment regimens.

## Abbreviations

BMI: Body mass index
CGMS: Continuous glucometer monitoring system
DM: Diabetes mellitus
G6P: Glucose-6 phosphate
GSD: Glycogen storage disease
